# Supplementary material for: A programmed cell division delay preserves genome integrity during natural genetic transformation in Streptococcus pneumoniae
Source: Nat Commun. 2017 Nov 20;8:1621. doi: 10.1038/s41467-017-01716-9 (PMC5696345; doi:10.1038/s41467-017-01716-9)
Supplement: Supplementary file 1 — Supplementary Information [file 41467_2017_1716_MOESM1_ESM.pdf]

Supplementary Note 1

ALL CELLS DEVELOP X-STATE (COMPETENCE)

Early studies suggested that in a *S. pneumoniae* population, 100 per cent of individual cells may be competent (i.e., have the capacity to absorb DNA) under optimal conditions. Such estimates were based on methods designed to exclusively register the irreversible uptake of DNA: (I) comparisons of single and double transformants frequencies for two unlinked markers[1,2]; or (II) calculation of the frequency of bacteria able to incorporate radioactive DNA as determined by autoradiography[3]. To assess the fraction of cells developing competence in pneumococcal cultures in a more rigorous manner, we directly visualized competence gene expression in individual cells by fluorescence microscopy using GFP (Green Fluorescent Protein) as a reporter. The *gfp* gene was placed under the control of P_x, a ComX-dependent promoter allowing GFP expression as a late *com* gene product[4]. This construct was introduced by transformation into the *S. pneumoniae* strain R895 at the CEP (<u>c</u>hromosomal <u>e</u>xpression <u>p</u>latform) locus[5]. The resulting strain (R3956, referred to as CEP_x-*gfp*), also carried a transcriptional fusion of the gene encoding luciferase (*luc*) with the *ssbB* gene, which is known to be specifically induced at competence. As expression of *ssbB::luc* is directly correlated with the kinetics of transformation in *S. pneumoniae*[6], this fusion enables monitoring of competence development at the level of the entire population through light emission by luciferase[7]. Cells were grown in C+Y medium as described in the *Methods* section and induced to develop competence by CSP addition. Samples were collected at different time points and analysed by fluorescence microscopy (Supplementary Fig. 1). Shortly after CSP addition, fluorescence intensity above background levels was detected in nearly all cells (Supplementary Fig. 1a). As an example, Supplementary Figure 1b shows an image captured 20 minutes after CSP induction in which all cells express GFP, indicating that competence develops in the entire culture. Although fluorescence was broadly heterogeneous, suggesting cell-to-cell variation of P_x:*gfp* expression, the population exhibited a gradual increase in fluorescence levels over time, culminating at maximal levels 40 minutes after competence induction and followed by a slow decrease (Supplementary Fig. 1c). Importantly, the curve pattern reported for fluorescence intensity is in good agreement with the competence profile obtained through measurement of luciferase activity (Supplementary Fig. 1c), although the maximum P_x:*gfp* expression level appears slightly delayed compared to that of *ssbB::luc*, presumably because of the slow rate of fluorescence acquisition of GFP[8]. In addition, the decrease of fluorescence is slower than the decrease of light emission, perhaps because the greater stability of GFP[9,10] results in its accumulation. The decrease in fluorescence intensity could thus reflect a dilution of the protein over cell division rather than a decrease of P_x:*gfp* expression.

We conclude from these experiments that all cells in pneumococcal cultures are able to develop competence. Similar observations have been made by others[11].

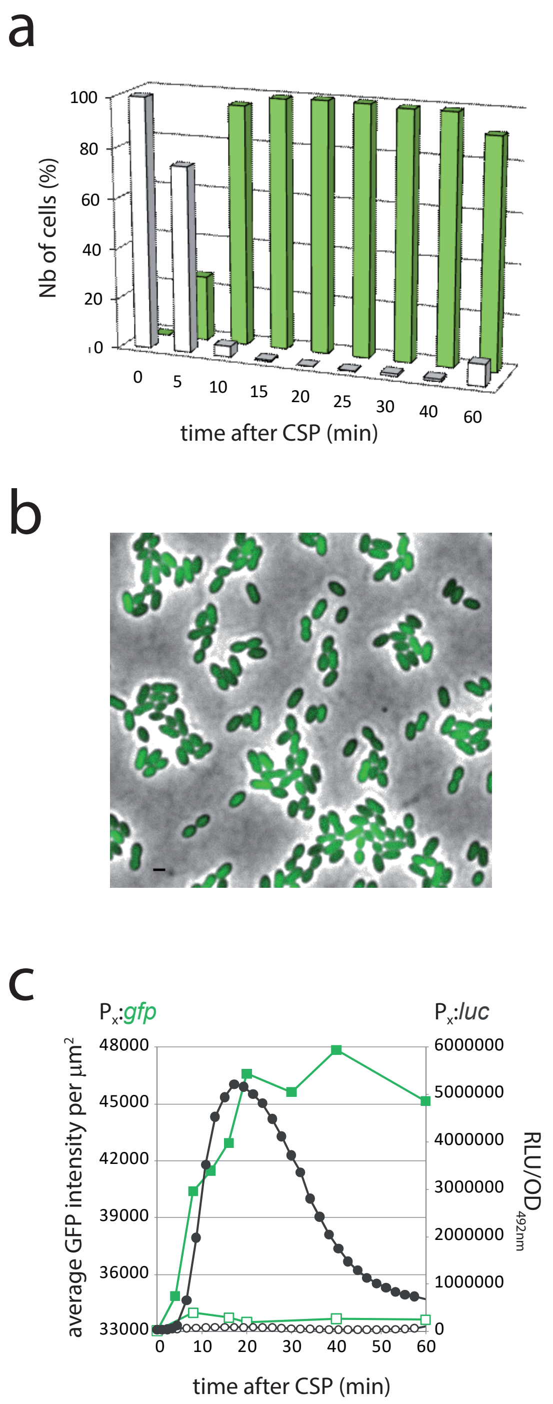

Supplementary Figure 1. All cells develop competence in pneumococcal culture. "Wild type" cells (strain R3956) were incubated with or without CSP. Placement of the *gfp* gene under the control of a ComX-dependent promoter allowed the study of single cells expressing GFP by microscopy. **a)** Histograms indicating the proportion of cells expressing GFP (in %). Average fluorescence intensity per μm^2 for individual cells was calculated as previously described[4]. A threshold value was determined by measuring the maximal fluorescence intensity emanating from autofluorescence in cells incubated without CSP. Cells exhibiting an average fluorescence intensity above the threshold value were counted as expressing GFP (*green bars*) and cells exhibiting a fluorescence intensity below this value were counted as not expressing GFP (*white bars*). Time after CSP induction is indicated in minutes. **b)** Fluorescence microscopy image showing cells expressing GFP 20 minutes after CSP addition. Overlay between phase contrast (*gray*) and GFP (*green*) is shown. *Scale bars*, 1 µm. **c)** Kinetics of competence induction measured with luciferase and GFP reporters. Expression of *gfp* (P_x:*gfp*) was monitored in cultures containing CSP (*green squares*) or not (*open green squares*). Expression of *ssbB*::*luc* (P_x:*luc*) was monitored in cultures containing CSP (*black circles*) or not (*open black circles*). Time after CSP induction is indicated in minutes. Data are representative of four independent experiments.

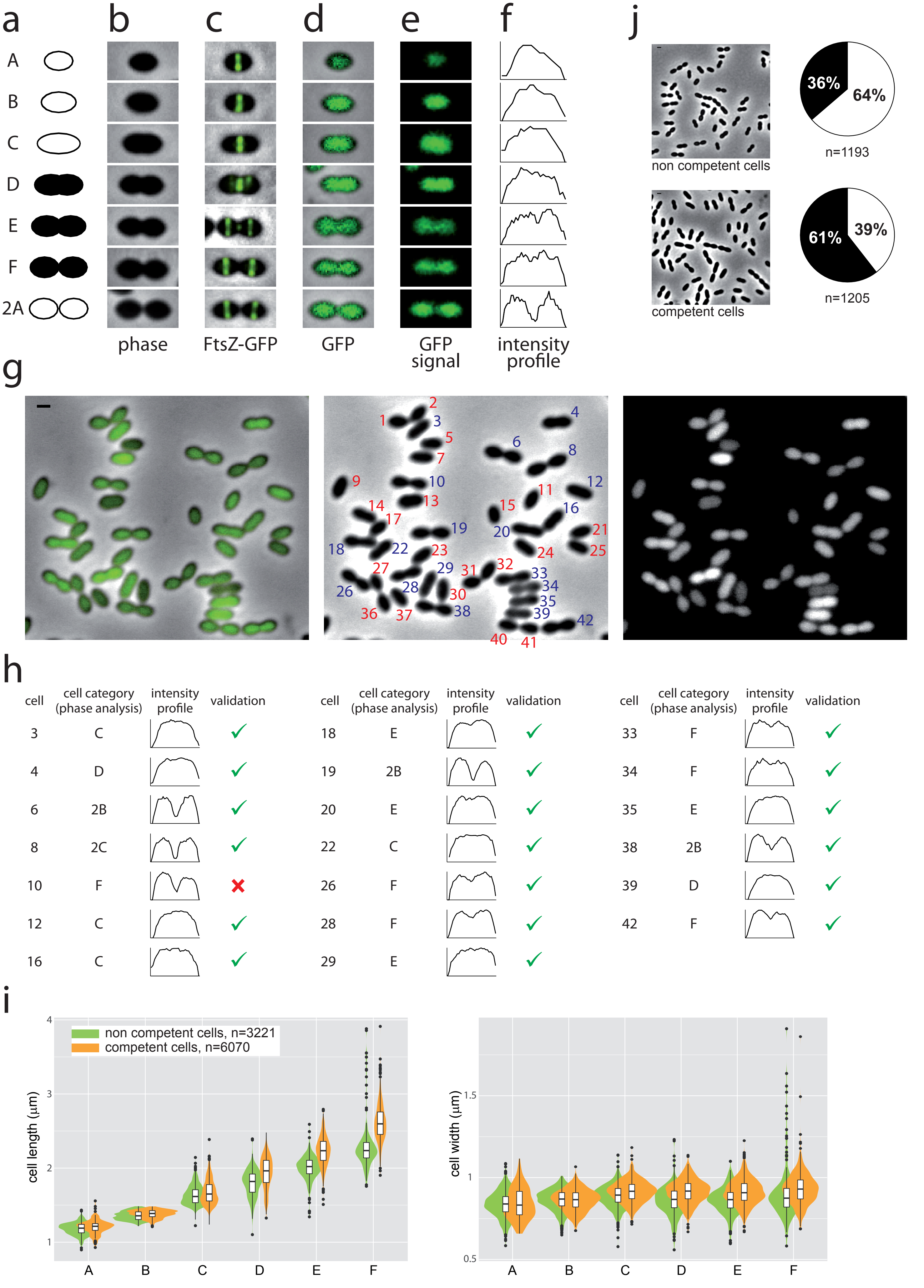

Supplementary Figure 2 . Classification of pneumococcal cells in different stages of the cell cycle.

Supplementary Figure 2. Classification of pneumococcal cells in different stages of the cell cycle. a-h) Discrimination between cells of categories F and newborn cells. **a)** Schematic representation of pneumococcal cells classified into 6 different classes according to the progression in their cell cycle. Dividing cells are represented in black. In these cells, including cells from category F, septum closure is not complete and the daughter cells cytoplasms are not compartmentalised. Completion of cell division in cells of category F gives rise to two newborn cells, depicted as two attached category A cells (2A). **(b)** Representative phase contrast images of cells for each category shown in *a*. **(c)** Representative images of cells harboring a FtsZ-GFP fusion for each category shown in *a*. Overlays between phase contrast (*gray*) and GFP (*green*) are shown. The FtsZ-GFP signal allows discrimination between cells of categories D, E and F. It cannot be used to distinguish cells of category F from newborn cells still attached (2A). **(d)** Representative images of cells producing GFP for each category shown in *a*. Overlays between phase contrast (*gray*) and GFP (*green*) are shown. **(e)** GFP signal shown in *d*. **(f)** GFP intensity profiles measured along the long axis of the cells shown in *d*. The x axis represents the cell length, and the y axis represents the fluorescence intensity (*arbitrary units*). The GFP intensity profiles allow discrimination between cells of categories F and newborn cells (2A). **(g)** Representative image of cells producing GFP. *Left*: Overlay between phase contrast (*gray*) and GFP (*green*). *Scale bar*, 1 µm. *Middle*: phase contrast image. Cells are numbered 1 to 42. *Right*: GFP signal. **(h)** Classification of cells marked with a blue number in *g*. The phase contrast image was used to classify the cells in each cell category shown in *a*, as described in the Methods section. GFP intensity profiles measured along the long axis of the cells are shown. These profiles were used to validate the classification deduced from the phase contrast image analysis. Analysis of the GFP intensity profiles revealed that the cell labelled with the number 10 in *g*, and predicted to belong to category F from the phase contrast analysis, corresponds to two newborn cells. The GFP intensity profiles of a total of 302 cells classified as cells of category F according to the phase contrast analysis were examined. Among these, only 20 were identified as newborn cells (6,6%), suggesting that our method of discriminating dividing cells from newborn cells is fairly accurate and reliable. **i)** Violin plots representing the cell size distribution of non competent and competent cells in the cell categories shown in *a*. *Left*: cell length distribution; *right*: cell width distribution. Boxes extend from the 25th percentile to the 75th percentile, with the horizontal line at the median. Whiskers represent the 95% confidence interval and dots represent outliers. n: number of cells analyzed. **j**) Encapsulated G54 cultures were induced to develop competence by CSP addition then incubated at 37°C for 30 minutes. *Left*: phase contrast images; *scale bars*, 1 µm. *Right*: pie charts show the distribution of non-dividing (*white*) and dividing (*black*) cells. n: number of cells analyzed. Cells were classified using the program developed to classify the cells of non-capsulated strains. Data are representative of 2 independent experiments.

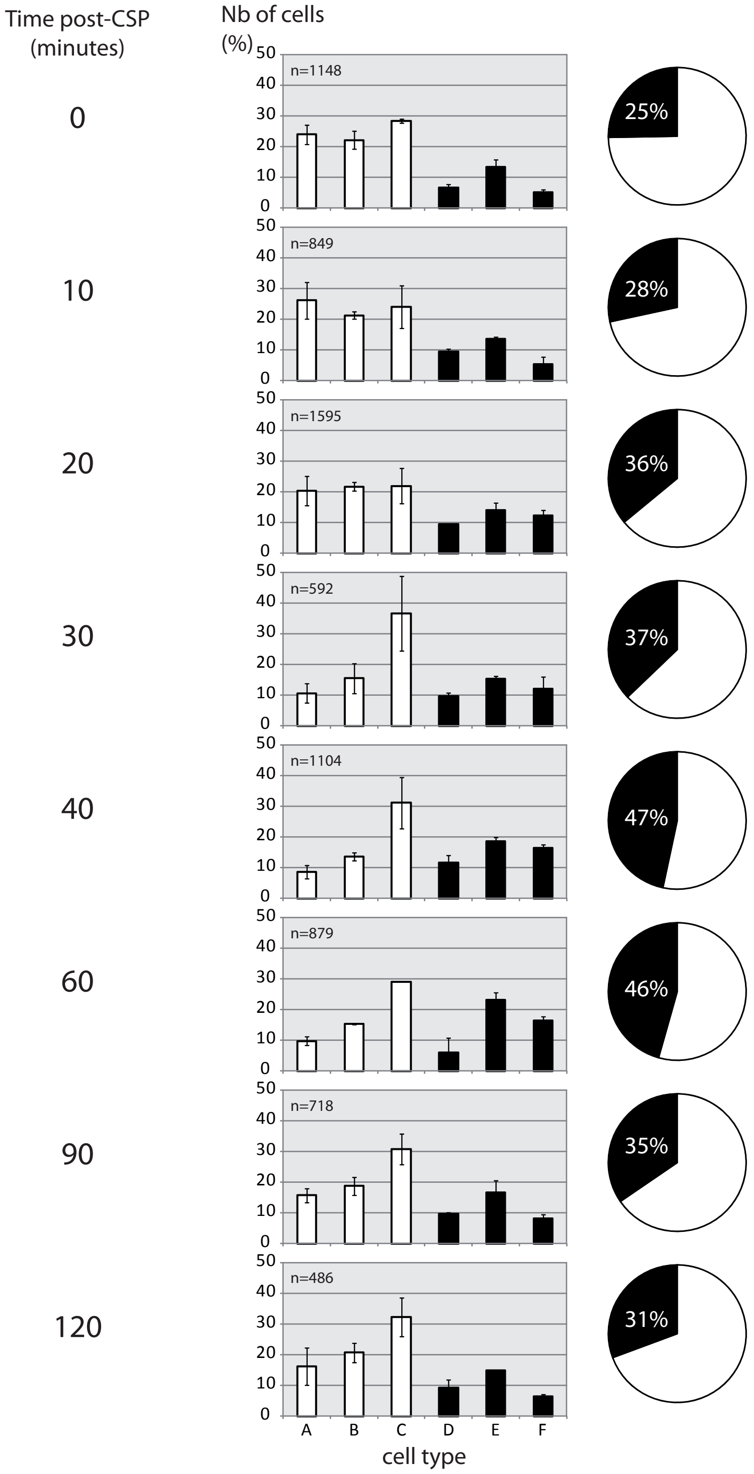

Supplementary Figure 3. Kinetics of the septation delay in wild type pneumococcal cultures developing competence. *ΔcomC* (R1501) "wild type" culture was induced to develop competence by CSP addition. At the times indicated, samples were taken and cells were imaged by phase contrast microscopy. Histograms indicate the percentage of cells in the cell type categories presented in Figure 1a (n: number of cells analyzed. Values and standard deviations are based on data from two independent experiments). Pie charts show the distribution of non-dividing (*white*) and dividing (*black*) cells.

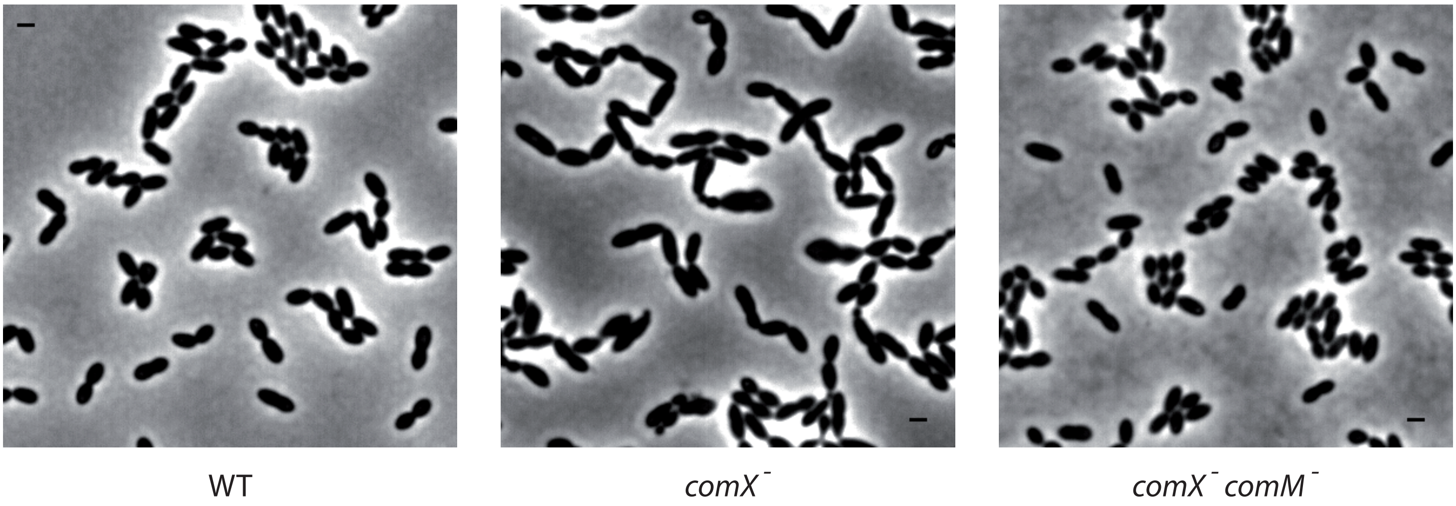

Supplementary Figure 4. Cell division defects persist in CSP-induced cells lacking ComX. Wild type (R1501, *left*), *comX*$^-$ (R2002, *middle*) and *comX*$^-$ *comM*$^-$ (R2132, *right*) cultures were treated with CSP and incubated at 37°C for 90 minutes. Samples were taken and cells were imaged by phase contrast microscopy. *Scale bar*, 1 µm. Images are representative of four biological replicates.

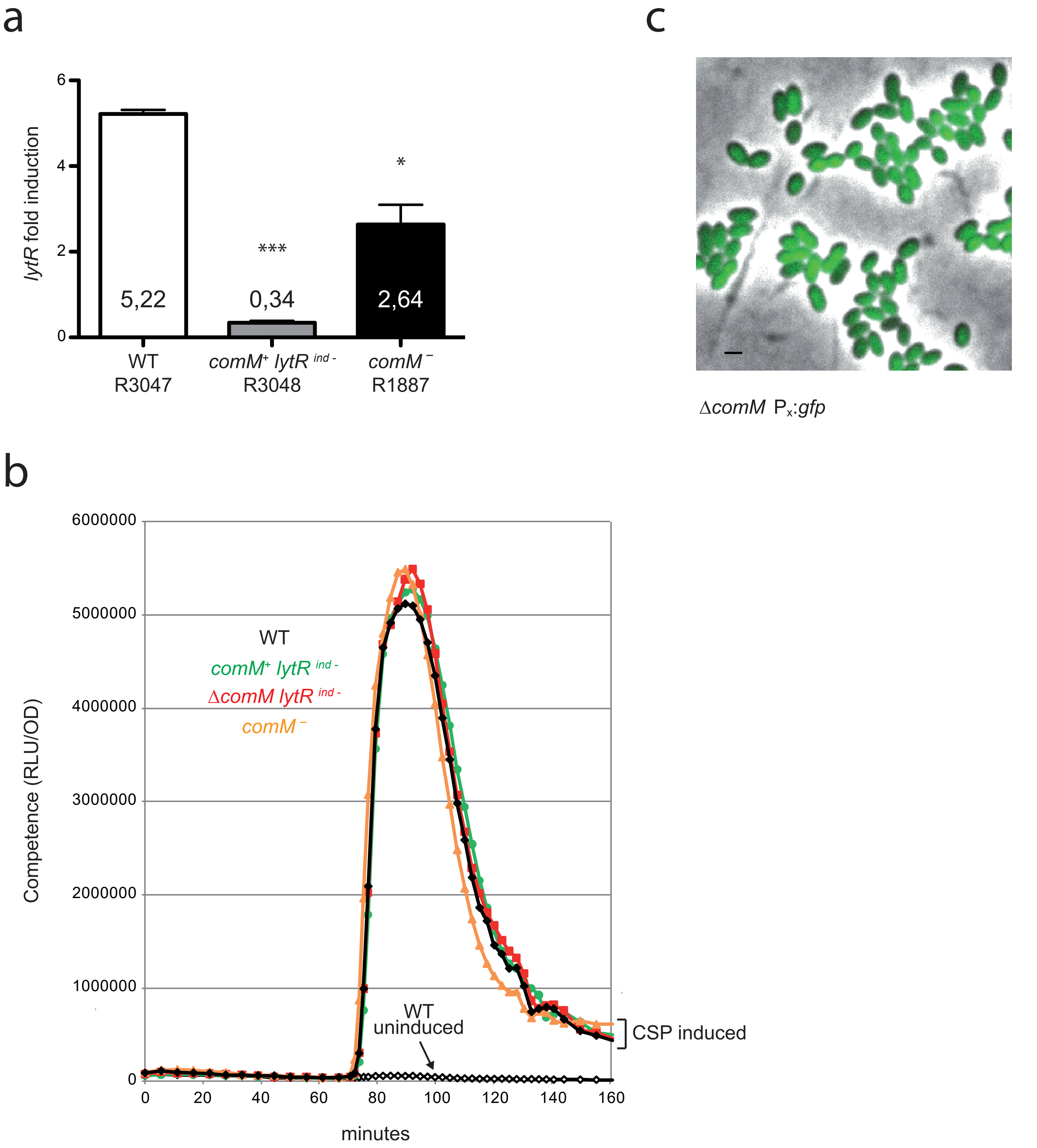

Supplementary Figure 5. Monitoring of competence induction. a) Induction of lytR mRNA levels in competent cultures relative to non competent cultures for wild-type (R3047), *comM*$^{+}$ *lytR*$^{ind-}$ (R3048), and *comM*$^{-}$ (R1887) strains. Induction of lytR transcripts were quantified in two independent experiments and 3 technical replicates using RTqPCR. Bars represent the mean and error bars represent the s.e.m. *, P=0.03; ***, P=0,0005 (two-tailed unpaired t-test versus WT). **b)** Expression of *ssbB::luc* transcriptional fusion was monitored in wild-type (R3047, *black symbols*), *comM*$^{+}$ *lytR*$^{ind-}$(R3048, *green symbols*), *ΔcomM lytR*$^{ind-}$ (R3049, *red symbols*) and *comM*$^{-}$ (R1887, *orange symbols*) cells following addition of CSP (*closed symbols*) or not (*open symbols*) after 70 minutes incubation at 37°C in C+Y medium. Data are representative of 3 independent experiments. **c)** GFP expression under the control of a ComX-dependent promoter (P_x) in individual *comM* mutant cells. R3989 (*ΔcomM* P_x:*gfp*) cells were analyzed by fluorescence microscopy 20 minutes after CSP addition. Overlay between phase contrast (*gray*) and GFP (*green*) is shown. *Scale bar*, 1 µm. Images are representative of four biological replicates.

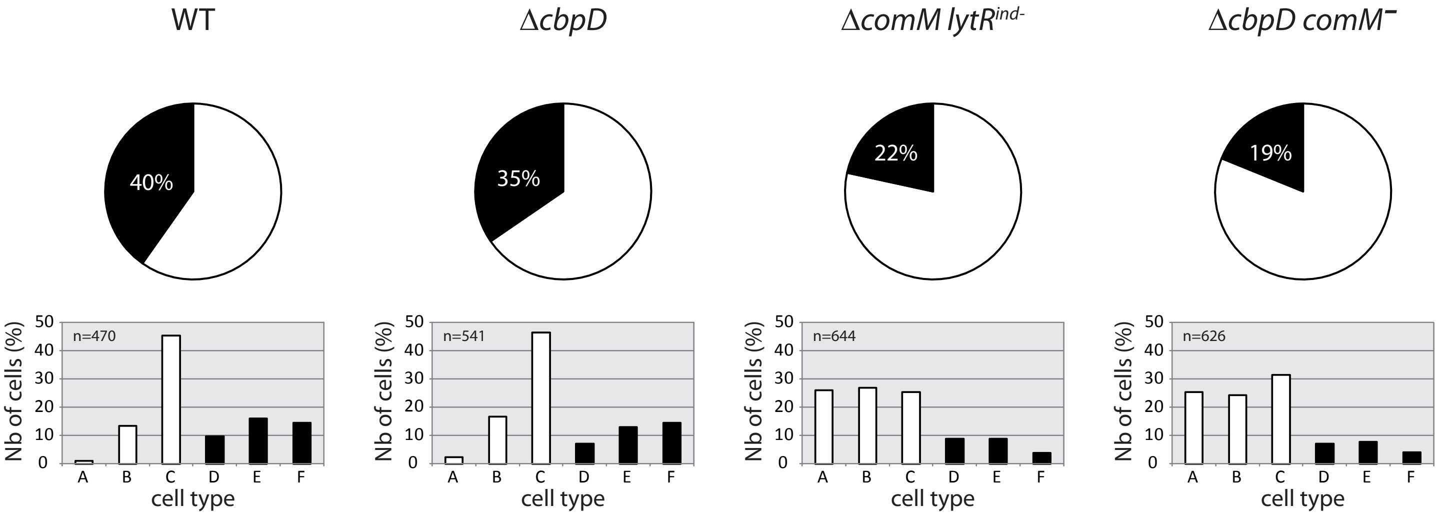

Supplementary Figure 6. Cell type distribution in competent *cbpD* mutant cells. Wild-type (R3047), Δ*cbpD* (R1720), Δ*comM lytR*$^{ind-}$ (R3049) and Δ*cbpD comM*$^{-}$ (R1886) cultures were induced to develop competence by CSP addition then incubated at 37°C for 30 minutes. Histograms indicate the percentage of cells in the cell type categories presented in Figure 1a (n: number of cells analyzed). Pie charts show the distribution of non-dividing (*white*) and dividing (*black*) cells. Data are from a single representative of 3 independent experiments.

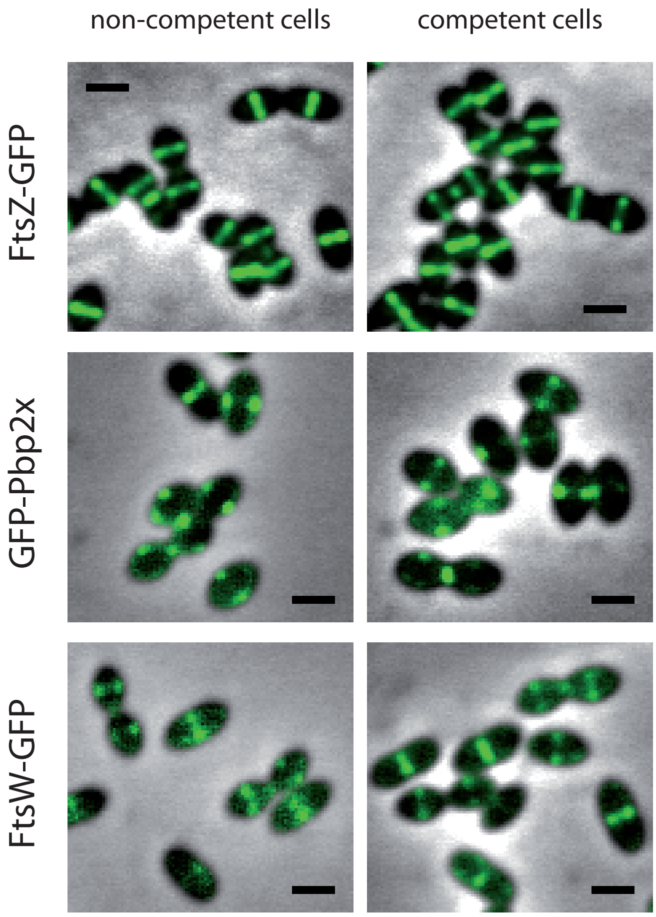

Supplementary Figure 7. Localisation of cell division proteins in non-competent and competent cells. Cultures were analyzed by fluorescence microscopy before (non-competent cells) and 30 minutes after CSP addition (competent cells). Strains used: R3702 (FtsZ-GFP); R3676 (GFP-Pbp2x) and R3677 (FtsW-GFP). Overlays between phase contrast (*gray*) and GFP (*green*) are shown. *Scale bars*, 1 µm. Images are representative of four independent experiments.

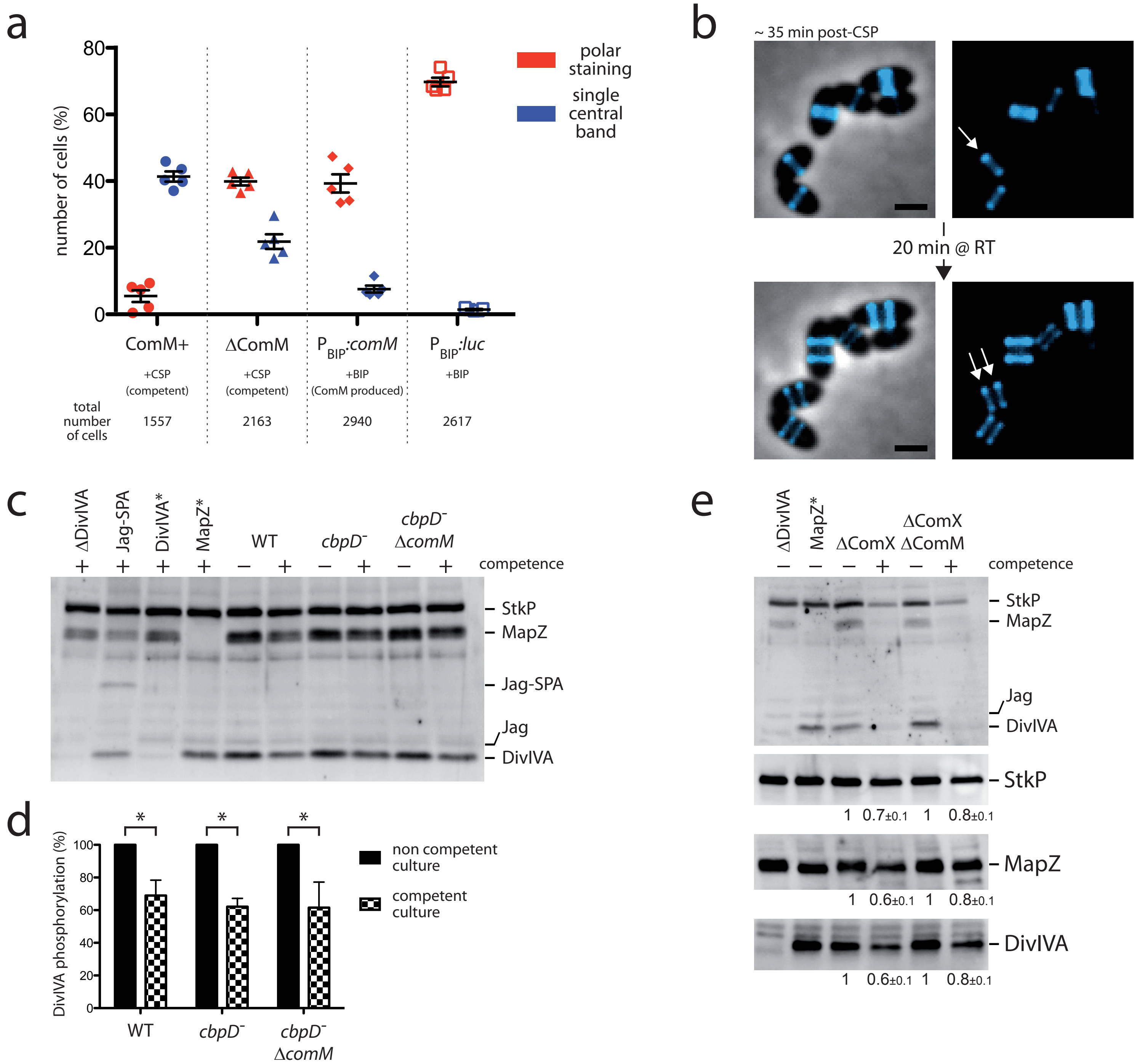

Supplementary Figure 8. Competence interferes with peptidoglycan synthesis and StkP kinase activity.
a) Peptidoglycan synthesis is negatively affected by the presence of ComM . Competent cells (+CSP) and cells treated with BIP (+BIP) were subjected to the same experimental protocol as in Fig. 5a,b. Graphic representation of the fraction of cells harboring polar HADA staining (*red*) and cells with a single thin HADA band at midcell (*bleu*). Strains used: ComM+ (R3966), ΔComM (R3967), P_{BIP}:*comM* (R3957) and P_{BIP}:*luc* (R2524). Mean and s.e.m. were calculated using the data from two and three technical replicates of two independent experiments. The total number of cells analyzed is indicated. **b)** Outcome of HADA single central band staining. Cells induced to develop competence for 10 minutes were incubated for 15 minutes with HADA, washed and spotted on a microscope slide covered with a pad of nutrient-agarose (~35 min post-CSP). Images were taken at an interval of 20 minutes, during which cells were allowed to grow in the microscope chamber at room temperature (RT). *Left*: false colored overlay images (phase contrast, *gray*; HADA, *blue*); *right*: fluorescent images. *Scale bars*, 1 μm. Images are representative of two biological replicates. **c-d)** Western blot of cell lysates probed with anti-phosphothreonine antibodies. Samples were prepared from competent (+) or not competent (-) cultures. Control strains harboring a deletion of the *divIVA* gene (R4134), a Jag-SPA fusion (R4143) and phosphoablative mutants of DivIVA (R4144) and MapZ (mapZ-2TA) were used to distinguish the phosphorylation signals for StkP, MapZ, Jag and DivIVA. **(c)** Biological replicate of experiment shown in Fig. 5c. Strains used: WT (R1501), *cbpD*- (R3966), *cbpD*- Δ*comM* (R3967). Full blot is shown in Supplementary Fig. 12c. Data are representative of seven biological replicates and 3 technical replicates. **(d)** Quantification of DivIVA phosphorylation. Each value represents the mean ±s.e.m. of three independent experiments. Two-way anova followed by Bonferroni multiple comparison revealed significant differences for non competent versus competent cultures (*, P<0,05). **(e)** *Top* panel: Biological replicate of experiment shown in Fig. 5d. Strains used: ΔComX (R2002), ΔComX ΔComM (R2132). *Lower* panels: protein levels of StkP, MapZ and DivIVA. Relative levels for competent versus non competent cultures are indicated. Values represents the mean ±s.e.m. for two biological replicates and 1 technical replicate. Full blots are shown in Supplementary Fig. 12d,e.

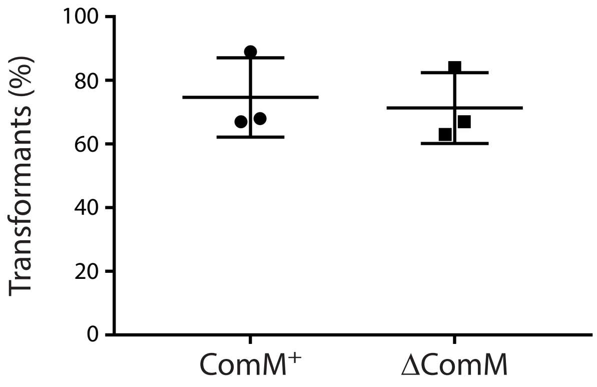

Supplementary Figure 9. Transformation frequency with a PCR fragment as donor DNA. Transformation with a 4.2-kb PCR fragment containing the *rpsL41* mutation conferring resistance to streptomycin as donor (200 ng mL^{-1}). Recipient strains: ComM+ (R3966), ΔComM (R3967). Mean and s.d. were calculated using the data from three independent experiments. Two-tailed paired t-test indicated no significant difference (P= 0,1835).

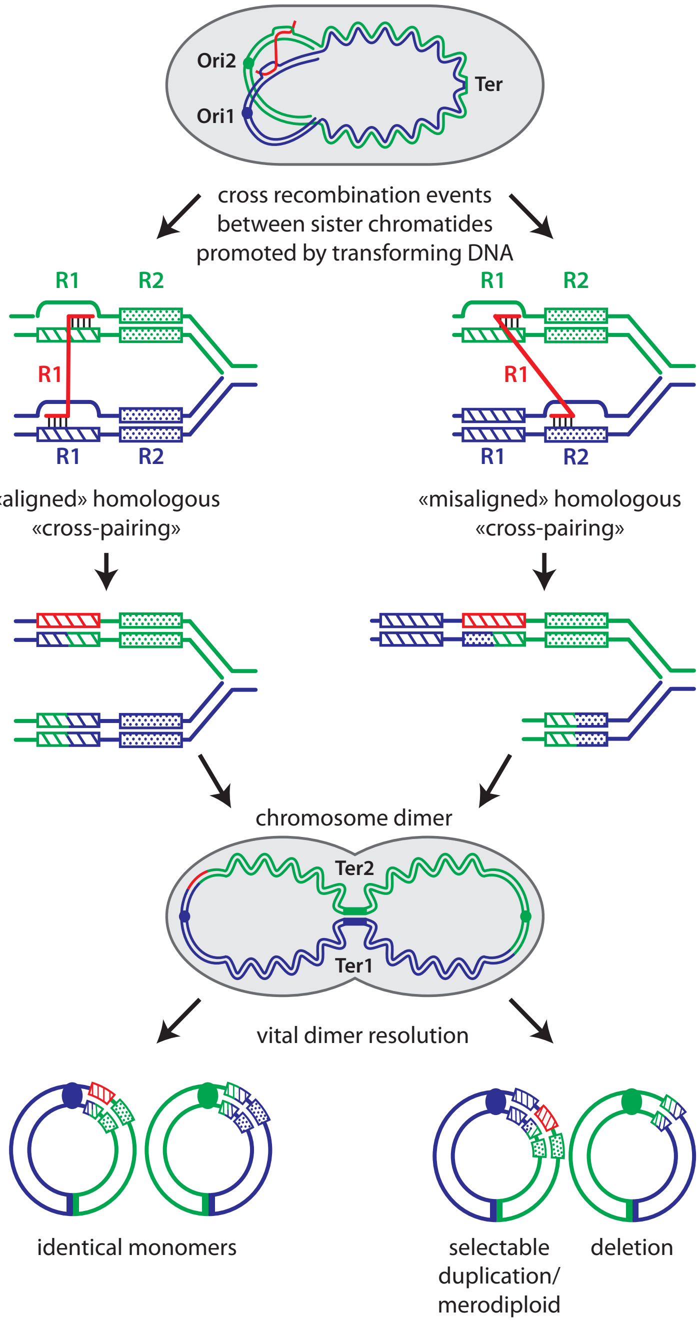

Supplementary Figure 10. Creation of chromosome dimers by transformation. "Aligned" homologous pairing of a donor DNA (*red*) carrying a sequence repeated at two sister loci of a partially replicated chromosome produces a chromosome dimer subsequently resolved into two identical monomers (*left*). "Misaligned" homologous pairing of part of the donor DNA with different copies of a repeat sequence carried by two daughter chromatids results in formation of a chromosome harboring a tandem duplication (merodiploid strain) and another chromosome with a deletion (potentially lethal).

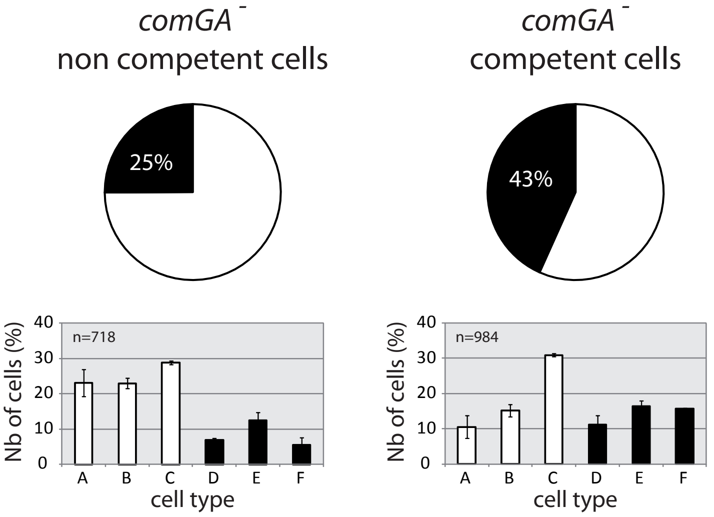

Supplementary Figure 11. ComGA is not required for cell division delay in pneumococcal competent cultures. *comGA*⁻ (R1916) cultures were induced to develop competence by CSP addition then incubated at 37°C for 30 minutes. Samples were taken and cells were imaged by phase contrast microscopy. Histograms indicate the percentage of cells in the cell type categories presented in Figure 1a (n: number of cells analyzed). Pie charts show the distribution of non-dividing (*white*) and dividing (*black*) cells. Values and standard deviations are based on data from three independent experiments.

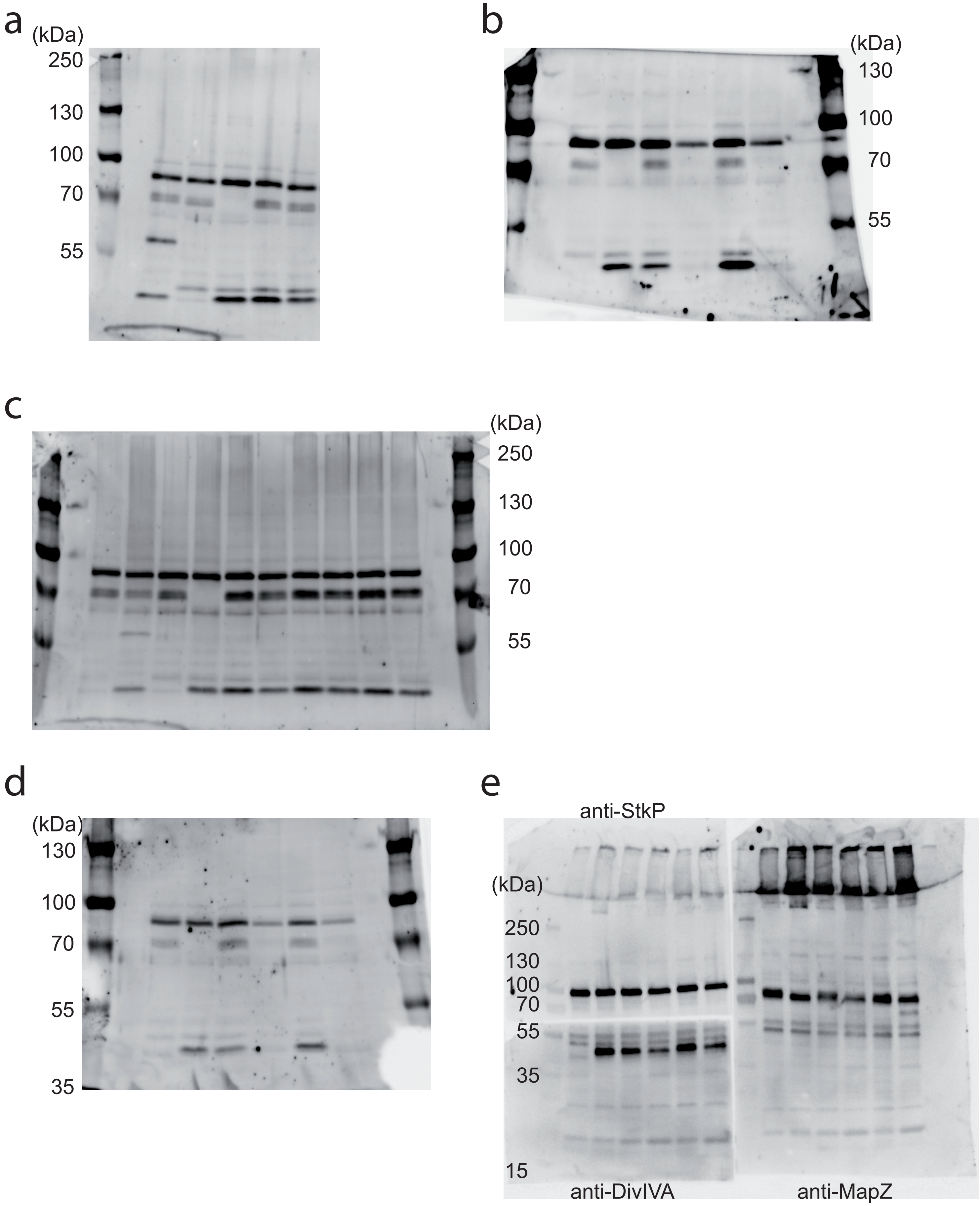

Supplementary Figure 12. Unaltered western blots used in manuscript. a) Blot related to Fig. 5C. **b)** Blot related to Fig. 5d. **c)** Blot related to supplementary Fig. 8c. **d)** Blot related to top panel of supplementary Fig. 8e. **e)** Blots related to lower panels of supplementary Fig. 8e.

Supplementary Methods

Plasmid and strain constructions

To construct strains R3047, R3048 (*comM*$^{+}$ *lytR*$^{ind-}$) and R3049 (*ΔcomM lytR*$^{ind-}$), the *kan-rpsL*+ Janus cassette[12] was first inserted downstream the *comM* gene and immediately upstream of Pc, the constitutive -10 extended promoter controlling the expression of the *spr1761*, *spr1760* and *lytR* genes (see Fig. 3). For this, strain R2995 was generated as follows. Plasmid pGBDU-*comM* was constructed to introduce the *Spe*I and *Bam*HI restriction sites at the chromosomal site of the Janus insertion. Primers were designed to amplify PCR products containing: (I) the 3' region of the *plcR* gene and the entire *comM* gene (1791bp; oligonucleotides comM1 and comM2, and R304 DNA as template); and (II) the Pc promoter together with the 5' region of the *spr1761* gene (470bp; oligonucleotides comM3 and comM4, and R304 DNA as template). The PCR products were gel-purified and used as templates in a SOEing PCR using the outer primers comM1 and comM4. The resulting SOEing PCR product was subsequently cut with *Eco*RI and *Cla*I, and ligated with the pGBDU plasmid[13] also cut with *Eco*RI and *Cla*I. To introduce the Janus cassette at the *Spe*I/*Bam*HI site a *kan-rpsL*$^{+}$ PCR product (oligonucleotides kan5spe and 7bam, and R1029 DNA[12] as template) was cut with *Spe*I and *Bam*HI and ligated with pGBDU-*comM* also cut with *Spe*I and *Bam*HI. Finally strain R2995 was created by using this ligation mixture to directly transform R981 pneumococcal cells[12] and select for clones sensitive to streptomycin. Plasmid pGBDU-*comM*-T1T2 was created to introduce the strong transcription terminators of *E. coli rrnB*, T1T2, immediately upstream of the Pc promoter. The activity of this terminator has been previously observed in pneumococcal strains[4]. A PCR product carrying the terminators (226bp; oligonucleotides T1T2spe and T1T2bam, and plasmid pUC57-T1T2*comC*[4] as template) was cut with *Spe*I and *Bam*HI and ligated into the pGBDU-*comM* plasmid also cut with *Spe*I and *Bam*HI. In parallel, plasmid pGBDU-Δ*comM* was constructed to delete the entire *comM* gene including its ComE-dependent promoter by cutting plasmid pGBDU-*comM* with *Nhe*I and *Spe*I and recircularising the 7425bp *Nhe*I/*Spe*I fragment by ligation. Finally, strains R3047, R3048 and R3049 were generated by transforming strain R2995 with plasmids pGBDU-*comM*, pGBDU-*comM*-T1T2 and pGBDU-Δ*comM* respectively and selecting for clones resistant to streptomycin. Resulting strains were subsequently transformed with genomic DNA from strain R895 to transfer the *ssbB::luc* (*ssbB*$^{+}$, *cat*) construct.

To achieve ectopic expression of *comM* in strain R3957, we constructed a derivative of pCEP$_{R}$, an integrative plasmid that allows integration of a gene at the CEP chromosomal expression platform[5] and its expression under the control of the P$_{R}$ promoter inducible by the BIP-1 peptide[14], also called P$_{BIP}$ for simplicity. Plasmid pNC73 (i.e., pCEP$_{R}$-*comM,* Kan^{R}) was created by inserting a *Nco*I-*Bam*HI PCR product containing the RBS and the orf of the *comM* gene (oligonucleotide

OCN64 and OCN65, and R304 DNA as template) into the $pCEP_R$ plasmid[14] cut with *Nco*I and *Bam*HI.

Strain R3966, harboring the markerless *cbpD*^{C75A} mutation, was generated using a PCR fragment amplified from strain R1902[15] using cbpD10 and cbpD11 as primers. The PCR product was used to transform the *S. pneumoniae* strain R1501 without selection as previously described[16]. Presence of mutation C75A in *cbpD* was confirmed by sequencing the *cbpD* gene.

Strain R3983, carrying a *gfp-comM* fusion at the *comM* endogenous locus, was obtained by transformation of strain R1501 with plasmid pCN83 and without selection as follows. Plasmid pCN72 ($pCEP_E$-*gfp-comM*, Kan^R) was first created in a three-way ligation with an *Nco*I-*Xho*I PCR product containing the *gfp* gene with codons optimized for *S. pneumoniae* (oligonucleotides OMB2 and OCN39, and template DNA pUC57-*gfp*(*Sp*)[4]), a *Xho*I-*Bam*HI PCR product containing the *comM* orf (oligonucleotides OCN63 and OCN64, and R304 DNA as template), and $pCEP_E$[17] cut with *Nco*I and *Bam*HI. pCN81 results from the replacement of the *Eco*RI-*Nco*I fragment from plasmid pCN72, carrying the ComE-dependent promoter, by a *Eco*RI-*Nco*I PCR product containing the region upstream of *comM* (1250 bp; primer pair OCN72 and OCN73, and R304 DNA as template). Finally, to generate plasmid pCN83, primers were designed to amplify the *comM* orf and a region downstream of *comM* (1300 bp; primer pair OCN63 and OCN74, and R304 DNA as template). The PCR product was subsequently cut with *Xho*I and *Spe*I, and ligated with the pCN81 plasmid also cut with *Xho*I and *Spe*I.

Strain R3676, harboring a *gfp-pbp2x* fusion at the *pbp2x* endogenous locus, was generated as follows. Plasmid pCN75 ($pCEP_M$-*gfp-pbp2x*, Kan^R) was first created in a three-way ligation with an *Nco*I-*Xho*I PCR product containing the *gfp* gene with codons optimized for *S. pneumoniae* (oligonucleotides OMB2 and OCN39, and template DNA pUC57-*gfp*(*Sp*)[4]), a *Xho*I-*Bgl*II PCR product containing the *pbp2x* orf (2253 bp; oligonucleotides OCN59 and OCN60, and R304 DNA as template), and plasmid $pCEP_M$[5] cut with *Nco*I and *Bam*HI. This ligation mixture was directly used for transformation of strain R1501 selecting for clones resistant to kanamycin. The resulting strain was used as a template to amplify a PCR fragment containing the *gfp-pbp2x* construct using oligonucleotides amiF1 and OCN60. The *gfp-pbp2x* fragment was subsequently cut with *Nco*I and *Bgl*II and ligated with a *Eco*RI-*Nco*I PCR fragment containing the region upstream of *pbp2x* (1550 bp; primer pair OCN70 and OCN71, and R304 DNA as template) into plasmid pUC57 cut with *Eco*RI and *Bam*HI. Finally, this ligation mixture was directly used for transformation of strain R1501 without selection, as previously described[16].

Strain R3677, containing a *ftsW-gfp* fusion at the *ftsW* endogenous locus, was created as follows. Plasmid pCN82 (pUC57-*ftsW-gfp*, Amp^R) was first generated in a three-way ligation with an *Eco*RI-*Xho*I PCR product containing the *ftsW* gene (1350 bp; oligonucleotides OCN57 and OCN58, and R304 DNA as template), a *Xho*I-*Bam*HI PCR product containing the *gfp* gene with codons optimized for *S. pneumoniae* (primer pair OMB4 and kan1, and plasmid pCN35[4] as template), and plasmid pUC57 cut with *Eco*RI and *Bam*HI. Plasmid pCN82 was subsequently cut open with *Bam*HI and *Hind*III and ligated with a *Bam*HI/*Hind*III PCR fragment containing the region downstream ftsW (1265bp; oligonucleotides OCN68 and OCN69, and R304 DNA as template). This ligation mixture was directly used for transformation of strain R1501 without selection, as previously described[16].

Strain R4134, containing a deletion of the gene encoding for DivIVA (*spr1505)*, was generated as follows. Primers were designed to amplify PCR products containing: (I) the upstream region of the *divIVA* gene (1013 bp; oligonucleotides OCN109 and OCN110, and R304 DNA as template); (II) the gene *aad9* conferring spectinomycin resistance (810 bp; oligonucleotides OCN111 and OCN112, and plasmid DNA pR412[18] as template); and (III) the downstream region of the *divIVA* gene (116 bp; oligonucleotides OCN113 and OCN114, and R304 DNA as template). The PCR products were gel-purified and used as templates in a SOEing PCR using the outer primers OCN109 and OCN114. The resulting SOEing PCR product was subsequently used to transform strain R895.

Strain R4143, containing a *jag-SPA* fusion, was generated as follows. Primers were designed to amplify PCR products containing: (I) the upstream region and the entire coding sequence of gene *spr1851* (*jag*[19] also called *EloR*[20]) (1183 bp; oligonucleotides OCN167 and OCN168, and R304 DNA as template); (II) the sequence encoding the sequential peptide affinity (SPA) tag[21] (276 bp; oligonucleotides OCN169 and OCN170, and plasmid pMutin-SPA[22] as template); and (III) the downstream region of the *spr1851* gene *(*1397 bp; oligonucleotides OCN171 and OCN166, and R304 DNA as template). The PCR products were gel-purified and used as templates in a SOEing PCR using the outer primers OCN167 and OCN166. The resulting SOEing PCR product was subsequently used to transform strain R1501 without selection, as previously described[16].

Strain R4144, which contains the phosphoablative form of DivIVA ($DivIVA^{T201A}$), was generated using a PCR fragment amplified from strain *divIVA-T201A*[23] using OCN176 and OCN179 as primers. The PCR product was used to transform the *S. pneumoniae* strain R1501 without selection as previously described[16]. Presence of mutation T201A in *divIVA* was confirmed by sequencing the *divIVA* gene.

Fluorescence microscopy and analysis

After gentle thawing of stock cultures, aliquots were inoculated at OD_{550} 0.006 in C+Y medium and grown at 37°C to an OD_{550} of 0.3. These precultures were inoculated (1/100) in C+Y medium and incubated at 37°C to an OD_{550} of 0.06. Then, competence was induced with synthetic CSP1 (25 ng ml^{-1}). At different times after CSP addition, 1 ml samples were collected, cooled down by addition of 500 µl cold C+Y medium, pelleted (3 min, 3,000 *g*) and resuspended in 50 µl C+Y medium. Two µl of this suspension was spotted on a microscope slide containing a slab of 1.2% C+Y agarose as described previously[24]. For time-lapse microscopy experiments (Fig. 1e), cells grown at 37°C to an OD_{550} of 0.1, were induced with CSP1 (100 ng ml^{-1}) for 2 minutes and 0.5 µl was immediately spotted on a microscope slide containing a slab of 1.2% C+Y agarose.

To analyze the cell type repartition of cells synthesizing ComM outside competence development, strains R2524 and R3957 were inoculated at OD_{550} 0.006 in C+Y medium and grown at 37°C to an OD_{550} of 0.3. These precultures were inoculated (1/100) in C+Y medium and incubated at 37°C to an OD_{550} of 0.06. *luc* or ectopic *comM* expression were induced by 250 µg ml^{-1} BIP-1. In parallel, endogenous *comM* expression was induced with 25ng ml^{-1} CSP1. Cells were incubated a further 30 min at 37°C. 1 ml samples were collected and treated as above before image acquisition.

Phase contrast and fluorescence microscopy were performed with an automated inverted epifluorescence microscope Nikon Ti-E/B equipped with the “perfect focus system” (PFS, Nikon), a phase contrast objective (CFI Plan Fluor DLL 100X, NA1.3), a Semrock filter set for GFP (Ex: 482BP35; DM: 506; Em: 536BP40), a Nikon Intensilight 130W High-Pressure Mercury Lamp, and a monochrome OrcaR2 digital CCD camera (Hamamatsu). The time-lapse microscopy experiment shown in Figure 1e was performed with a similar Nikon Ti-E/B microscope equipped with the PFS, a phase contrast objective (CFI Plan Apo Lambda DM 100X, NA1.45), a Semrock filter set for GFP (Ex: 482BP35; DM: 506; Em: 536BP40), a LED light source (Spectra X Light Engine, Lumencor), and a sCMOS camera (Neo sCMOS, Andor). Both microscopes are equipped with a chamber thermostated at 30°C and adjusted to 37°C when required. Fluorescence images were captured and processed using Nis-Elements AR software (Nikon). GFP fluorescence images were false colored green and overlaid on phase contrast images.

Supplementary Table 1

Strain	Genotype/ relevant feature[a]	Reference	Figure
R800	*Wild-type* (R6 derivative)	[25]	
R304	*nov1, rif23, str41;* Nov^R, Rif^R, Strep^R	[26]	
R895	*ssbB::luc* (*ssbB*$^+$, *cat*); Cat^R	[27]	
R981	*rpsL1,* Strep^R	[12]	
R1029	Δ*comC::kan-rpsL*$^+$, Kan^R	[12]	
R1501	Δ*comC*	[28]	5c; 6; S3; S4; S8c,d
R1720	Δ*comC*, *cbpD::kan*, *ssbB::luc* (*ssbB*$^+$, *cat*); Kan^R, Cat^R	Lab collection[b]	S6
R1879	Δ*comC*, *comM::spec*, *ssbB::luc* (*ssbB*$^+$, *cat*); Spec^R, Cat^R	[15]	
R1886	Δ*comC*, *comM::spec*, *cbpD::kan*, *ssbB::luc* (*ssbB*$^+$, *cat*); Spec^R, Kan^R, Cat^R	Lab collection	S6
R1887	Δ*comM::spec*, *ssbB::luc* (*ssbB*$^+$, *cat*); Spec^R, Cat^R	Lab collection	3; S5a,b
R1902	*ssbB::luc* (*ssbB*$^+$, *cat*), *hexA::ermAM*, *comA::kan*, *cpbD*^{C75A}; Cat^R, Ery^R, Kan^R	[15]	
R1916	Δ*comC*, *ssbB::luc* (*ssbB*$^+$, *cat*), *comGA::kan*; Cat^R, Kan^R	Lab collection[c]	S11
R2002	Δ*comC*, *comC::luc* (*comC*$^+$, *cat*), *comX1::ermAM*, *comX2::tet*; Cat^R, Ery^R, Tet^R	[29]	2; 5d; S4; S8e
R2132	Δ*comC*, *comM::spec*, *comX1::ermAM*, *comX2::tet*; Spec^R, Ery^R, Tet^R	Lab collection	5d; S4; S8e
R2524	Δ*comC*, CEP$_R$-*luc* (*kan*); Kan^R	[14]	4a; S8a
R2995	*comM*$^+$*-kan-rpsL*$^+$*-spr1760-spr1761-lytR, rpsL1*; Kan^R	this study	
R3047	*rpsL1*, *ssbB::luc* (*ssbB*$^+$, *cat*); Strep^R, Cat^R	this study	3; S5ab; S6
R3048	*comM*$^+$*-T1T2*$_{terminator}$*-spr1760-spr1761-lytR, rpsL1*, *ssbB::luc* (*ssbB*$^+$, *cat*); Strep^R, Cat^R (referred to as *comM*$^+$ *lytR*$^{ind-}$)	this study	3; S5a,b
R3049	Δ*comM-T1T2*$_{terminator}$*-spr1760-spr1761-lytR, rpsL1*, *ssbB::luc* (*ssbB*$^+$, *cat*); Strep^R, Cat^R (referred to as Δ*comM lytR*$^{ind-}$)	this study	3; S5b; S6
R3676	Δ*comC*, *gfp-pbp2x*	this study	S7
R3677	Δ*comC*, *ftsW-gfp*	this study	S7
R3702	Δ*comC*, *hexA*Δ*3::ermAM*, *ftsZ-gfp*; Ery^R	[30]	1e; S2c; S7
R3956	CEP$_x$-*gfp(Sp)* (kan) [from plasmid pCN35], *ssbB::luc* (*ssbB*$^+$, *cat*); Kan^R, Cat^R	this study	1; S1; S2
R3957	Δ*comC*, CEP$_r$-*comM* (*kan*) [from plasmid pCN73]; Kan^R	this study	4a; S8a
R3966	Δ*comC*, *cbpD*^{C75A}	this study	5a,b; 6; S8a,b,c,d; S9
R3967	Δ*comC*, *cbpD*^{C75A}, *comM::cat*; Cat^R	this study[d]	5a,b; 6; S8a,c,d; S9
R3970	Δ*comC*, *cbpD*^{C75A}, *recO::spec, xerS::kan;* Spec^R, Kan^R	this study	6
R3973	Δ*comC*, *cbpD*^{C75A}, *comM::cat, recO::spec, xerS::kan;* Cat^R, Spec^R, Kan^R	this study	6
R3983	Δ*comC*, *gfp-comM* [from plasmid pCN83]	this study	4b

R3989	Δ*comM-T1T2*$_{terminator}$-*spr1760-spr1761-lytR, rpsL1*, *cbpD::spec*, *ssbB::luc* (*ssbB*$^{+}$, *cat*), CEP$_{x}$-*gfp(Sp)* (kan) [from plasmid pCN35]; Strep^{R}, Spec^{R}, Cat^{R}, Kan^{R}	this study	S5c
R4134	*ssbB::luc* (*ssbB*$^{+}$, *cat*); *divIVA::spec*; Cat^{R}, Spec^{R}	this study	5d; S8c,e
R4143	Δ*comC*, *jag-SPA*	this study	5c; S8c
R4144	Δ*comC*, *divIVA*^{T201A}	this study	5c; S8c
mapZ-2TA	*rpsL1*, *mapZ*$^{T67A\text{-}T78A}$; Strep^{R}	[31]	5c,d; S8c,e

a: ^{R}, resistance ; Cat, chloramphenicol; Ery, erythromycin; Kan, kanamycin; Nov, novobiocin; Rif, rifampicin; Spec, spectinomycin; Strep, streptomycin; Tet, tetracyclin.
b: *cbpD*::*kan*: transformation with a PCR fragment amplified from the L3 strain[32] using cbpD1 and cbpD4 as primers[15].
c: *comGA::kan,* transformation with a PCR fragment containing a mariner cassette resulting in the co-transcribed insertion of a kanamycine resistance gene (position 652 with respect to the ATG of *comGA*).
d: *comM::cat,* the *spec mariner* cassette insertion in *comM* was exchanged with the *cat* cassette by transformation of strain R1866[15] with plasmid pEMCat DNA[33], selecting for Cat^{R} transformants. Cassette swapping is based on the presence of DNA homology at the borders of the synthetic *spec* and *cat* minitransposons, allowing exchange of resistance cassette genes by homologous recombination during transformation[34].
e: *recO::spec,* transformation with genomic DNA from strain R2372[35].
f: *xerS::kan,* transformation with genomic DNA from strain S501[36].

Supplementary Table 2

Plasmid	Description	Reference
pGBDU	pGBT9 derivative	[13]
pUC57	ColE1 derivative; 2710 bp; Amp^R	Genscript USA
$pCEP_E$	pCEP derivative containing instead of the maltose-driven promoter, P_M, the ComE-dependent promoter, P_E, of the *comCDE* operon and the RBS of *comC*; Kan^R, $Spec^R$	[17]
$pCEP_M$	pSC101 derivative carrying the chromosomal platform CEP and the maltose inducible promoter P_M; Kan^R	[5]
$pCEP_E$	pCEP derivative containing instead of the maltose-driven promoter, P_M, the ComE-dependent promoter, P_E, of the *comCDE* operon and the RBS of *comC*; Kan^R, $Spec^R$	[17]
$pCEP_R$	ColE1 (pBR322) derivative carrying the chromosomal platform CEP and the BlpR-dependent promoter, P_R; Kan^R	[14]
pUC57-*gfp*(*Sp*)	pUC57 derivative carrying a 728 bp *Nco*I-*Bam*HI synthetic fragment containing the *gfp*(*Sp*) gene encoding GFP with codon optimized for *S. pneumoniae* R6; Amp^R	[4]
pUC57-T1T2*comC*	pUC57 derivative carrying an 608 bp *Hind*III-*Bam*HI synthetic fragment with the T1T2 *E. coli* terminators; Amp^R	[4]
pCN35	ColE1 (pBR322) derivative carrying the chromosomal platform CEP and containing the *gfp*(*Sp*) gene encoding GFP with codon optimized for *S. pneumoniae* R6, under the control of the ComX-dependent promoter, P_X and the RBS of *ssbB*; Kan^R	[4]
pGBDU-*comM*	pGBDU derivative carrying a 2229 bp region containing the 3' part of the *plcR* gene, the *comM* gene and the 5' part of the *spr1761* gene with the *Spe*I and *Bam*HI restriction sites inserted downstream *comM* and upstream *spr1761*	this study
pGBDU-*comM*-T1T2	pGBDU-*comM* derivative carrying a *Spe*I-*Bam*HI 226bp fragment containing the T1T2 *E. coli* terminators; Amp^R	this study
pGBDU-Δ*comM*	pGBDU-*comM* derivative obtained by deletion of a *Nhe*I-*Spe*I 782 bp fragment containing the *comM* gene; Amp^R	this study
pCN72	$pCEP_E$ derivative containing a *gfp-comM* fusion; Kan^R	this study
pCN73	$pCEP_R$ derivative containing the *comM* gene under the control of the BlpR-dependent promoter, P_R; Kan^R	this study
pCN75	$pCEP_M$ derivative containing a *gfp-pbp2x* fusion; Kan^R	this study
pCN82	pUC57 derivative a *ftsW-gfp* fusion; Amp^R	this study
pCN83	Integrative plasmid allowing integration of the *gfp-comM* construct at the endogenous chromosomal locus without selection; Amp^R	this study

Supplementary Table 3

oligo	Sequence	Use
7bam	TTTGGATCCAGAGACCTGGGCCCCTTTCC	R2995
amiF1	GCCTTGCTTTCAGCGGTACCAAT	R3676
CbpD10	TGAGTCAGCTTTCTCGTGGTGTAG	R3966
CbpD11	AGACTAAGTATCGTTCCCTCCGCT	R3966
comM1	GCGGAATTCCCTCCGCAGACTTGTTAT	pGBDU-*comM*
comM2	GGATCCATAAGCACCACTAGTATAAAAAACAGGCTTTCTCTAAAAG	pGBDU-*comM*
comM3	ACTAGTGGTGCTTATGGATCCGTTATGATGATAAGTTTAGTAGG	pGBDU-*comM*
comM4	CGGATCGATCGGCGTCCATCTGCTTCTTTTAG	pGBDU-*comM*
Kan1	ATCATGTCCTTTTCCCGTTCCAC	pCN82
Kan5spe	CCCACTAGTGTTTGATTTTTAATGGATAATG	R2995
OCN39	CGACTCGAGTTTATACAATTCATCCATACCATGTG	pCN72, pCN75
OCN57	CAAGGACATATGAAGATTAGTAAGAGGCAC	pCN82
OCN58	GGTCTCGAGCTTCAACAGAAGGTTCATTGGT	pCN82
OCN59	GCTCTCGAGGGTTCCGGAATGAAGTGGACAAAAAGAGTAATCCGT	pCN75
OCN60	CGATAGATCTGTCACAATTCCAGCACTGATGGA	pCN75, R3676
OCN63	GCTCTCGAGGGTTCCGGAATGGAATCAATGAGAATCTTATTTTTGTTAG	pCN72, pCN83
OCN64	CCTGGATCCCTTATCATCATAACATCCA	pCN72, pCN73
OCN65	GGACCATGGTAGGAAGGGAGAGAGAAGA	pCN73
OCN68	GGCGGATCCGATAAAGAAAGGATAGTTTATGTCTCTTCA	R3677
OCN69	GGCAAGCTTCATAGACGCTAGAGTCTTGTCGCAT	R3677
OCN70	GCTGAATTCACACGATTCATCTGGTCTCCTTTCTGA	R3676
OCN71	CGCCCATGGCTTACTCCGCTATTCTAATATTTTCATTGTTTAATTGCA	R3676
OCN72	GAATGAATTCGAGACTCTCCCAACAAACTCTTGCAGAAGGA	pCN83, R3676
OCN73	CCTTCCATGGTCTCTCTCCCTTCCTACCAATCATTATACT	pCN83
OCN74	GACGGACTCTCTTACGCTGATCAGCTGTA	pCN83
OCN109	GGTAGCCTTCGCTGTCATGTCTTGGT	R4134
OCN110	GTATTCAAATATATCCTCCTCACTCACTTACTTAATAATAACTGGACGGT	R4134
OCN111	CCAGTTATTATTAAGTAAGTGAGTGAGGAGGATATATTTGAATACATACGA	R4134
OCN112	GACCTGTCGGATGCACTGGAGTTATAATTTTTTTAATCTGTTATTTAAATAGT	R4134
OCN113	TTAAATAACAGATTAAAAAAATTATAACTCCAGTGCATCCGACAGGTCCA	R4134
OCN114	CTGCACCAACCGTCAAACCACGAGA	R4134
OCN166	GCGTTGAGTTGCCTCAACAGTCA	R4143
OCN167	CCCTATACTGGACAGTGTCT	R4143
OCN168	ATCTCGAGTCGGCCGGAACCTTCTGTATCTACAACAACATAGCGAT	R4143
OCN169	ATCGCTATGTTGTTGTAGATACAGAAGGTTCCGGCCGACTCGAGAT	R4143
OCN170	ATCAGGATAAACCTGATTTTACTTACTACTTGTCATCGTCATCCTTGT	R4143
OCN171	ACAAGGATGACGATGACAAGTAGTAAGTAAAATCAGGTTTATCCTGAT	R4143
OCN176	CTATCTAGGAATCAAGCAAACCAGT	R4144
OCN179	CCCAGTCACCAGAAACACCCA	R4144
OMB2	GAATTCCCATGGTTTCTAAAGGTG	pCN72, pCN75

OMB4	AGACTCGAGGGTTCCGGAATGGTTTCTAAAGGTGAAG	pCN82
T1T2bam	GTGGGATCCAAAAAGGCCATCCGTCAGGATGGCCTTCTG	pGBDU-*comM*-T1T2
T1T2spe	TTTACTAGTTCAAATAAAACGAAAGGCTCAGTCG	pGBDU-*comM*-T1T2
OCN186	TGAAGCGACTGAACCTCTAACC	RTqPCR (*lytR*)
OCN187	GCTATCACTTCTACCGACCCAA	RTqPCR (*lytR*)
OCN188	GTTCAGCAGCTTCTTCTGTTGC	RTqPCR (*rpoB*)
OCN189	GGACGAAGATGTCATCCACG	RTqPCR (*rpoB*)

Supplementary Table 4

Cell Categorie[a]		Shape Factor[b]	Elliptical form Factor[c]	Length (µm)	Outer Radius (µm)[d]
A	A1	0.901 to 1	≤ 1.400	≤ 1.200	-
	A2	0.750 to 0,900	≤ 1.600	≤ 1.200	-
	A3	≥ 0.901	≥ 1.410	-	≤ 0.600
	A4	0.81 to 0.9	≥ 1.700	≤ 1.449	≤ 0.600
	A5	0.901 to 0.940	1.4001 to 1.4090	1.191 to 1.260	0.530 to 0.599
	A6	0.850 to 0.900	1.4001 to 1.6150	1.201 to 1.260	0.530 to 0.599
	A7	≥ 0.900	≤ 1.400	≥ 1.200	-
	A8	0.870 to 0.899	1,209 to 1.390	1.201 to 1.540	≥ 0.570
B	B1	≥ 0.801	≥ 1.410	-	0.600 to 0.700
	B2	0.760 to 0.809	1.500 to 1.590	1.200 to 1.560	0.569 to 0.699
	B3	0.760 to 0.809	1.500 to 1.590	1.201 to 1.299	0.569 to 0.760
	B4	0.810 to 0.869	≤ 1.400	≥ 1.200	≥ 0.5
C	C1	0.709 to 0.800	1.600 to 2.000	-	-
	C2	≥ 0,801	≥ 1.800	≥ 1.450	-
	C3	0.750 to 0.800	≥ 2.000	-	-
	C4	≥ 0.801	1.410 to 1.800	≥ 1.300	≥ 0.70
D	D1	0.708 to 0.749	≥ 2.000	-	0.600 to 0.700
	D2	0.600 to 0.708	1.800 to 2.000	-	-
E		0.600 to 0.708	≥ 2.000	-	-
F		≤ 0.600	≥ 2.000	-	-

[a], cell categories are divided in a set of subcategories (*i.e.*, categorie A includes subcategories A1 to A8).

[b], shape factor = $\frac{4\pi A}{P^2}$, where P is the perimeter and A, the area. A value near 0 indicates an elongated cell, whereas a value of 1.0 indicates a round cell.

[c], the ratio of the cell width to its length.

[d], the distance from the center of the cell to the farthest point along the cell's edge.

Supplementary References

1 Ephrussi-Taylor, H. in *Recent progress in microbiology (Intern. Congr. Microbiol., 7th, Stockholm, 1958)* Vol. III (ed G. Tunevall) 51-68 (Charles C Thomas, 1958).

2 Porter, R. D. & Guild, W. R. Number of transformable units per cell in Diplococcus pneumoniae. *Journal of bacteriology* **97**, 1033-1035 (1969).

3 Javor, G. T. & Tomasz, A. An autoradiographic study of genetic transformation. *Proceedings of the National Academy of Sciences of the United States of America* **60**, 1216-1222 (1968).

4 Martin, B. *et al.* Expression and maintenance of ComD-ComE, the two-component signal-transduction system that controls competence of Streptococcus pneumoniae. *Molecular microbiology* **75**, 1513-1528, doi:10.1111/j.1365-2958.2010.07071.x (2010).

5 Guiral, S. *et al.* Construction and evaluation of a chromosomal expression platform (CEP) for ectopic, maltose-driven gene expression in Streptococcus pneumoniae. *Microbiology* **152**, 343-349, doi:10.1099/mic.0.28433-0 (2006).

6 Berge, M., Moscoso, M., Prudhomme, M., Martin, B. & Claverys, J. P. Uptake of transforming DNA in Gram-positive bacteria: a view from Streptococcus pneumoniae. *Molecular microbiology* **45**, 411-421 (2002).

7 Prudhomme, M. & Claverys, J. P. *The Molecular Biology of Streptococci*. 519–524 (Horizon Bioscience, 2007).

8 Zimmer, M. Green fluorescent protein (GFP): applications, structure, and related photophysical behavior. *Chemical reviews* **102**, 759-781 (2002).

9 Chalfie, M., Tu, Y., Euskirchen, G., Ward, W. W. & Prasher, D. C. Green fluorescent protein as a marker for gene expression. *Science* **263**, 802-805 (1994).

10 Li, X. *et al.* Generation of destabilized green fluorescent protein as a transcription reporter. *The Journal of biological chemistry* **273**, 34970-34975 (1998).

11 Slager, J., Kjos, M., Attaiech, L. & Veening, J. W. Antibiotic-induced replication stress triggers bacterial competence by increasing gene dosage near the origin. *Cell* **157**, 395-406, doi:10.1016/j.cell.2014.01.068 (2014).

12 Sung, C. K., Li, H., Claverys, J. P. & Morrison, D. A. An rpsL cassette, janus, for gene replacement through negative selection in Streptococcus pneumoniae. *Applied and environmental microbiology* **67**, 5190-5196, doi:10.1128/AEM.67.11.5190-5196.2001 (2001).

13 James, P., Halladay, J. & Craig, E. A. Genomic libraries and a host strain designed for highly efficient two-hybrid selection in yeast. *Genetics* **144**, 1425-1436 (1996).

14 Johnston, C. *et al.* Fine-tuning of choline metabolism is important for pneumococcal colonization. *Molecular microbiology*, doi:10.1111/mmi.13360 (2016).

15 Havarstein, L. S., Martin, B., Johnsborg, O., Granadel, C. & Claverys, J. P. New insights into the pneumococcal fratricide: relationship to clumping and identification of a novel immunity factor. *Molecular microbiology* **59**, 1297-1307, doi:10.1111/j.1365-2958.2005.05021.x (2006).

16 Quevillon-Cheruel, S. *et al.* Structure-function analysis of pneumococcal DprA protein reveals that dimerization is crucial for loading RecA recombinase onto DNA during transformation. *Proceedings of the National Academy of Sciences of the United States of America*, doi:10.1073/pnas.1205638109 (2012).

17 Mirouze, N. *et al.* Direct involvement of DprA, the transformation-dedicated RecA loader, in the shut-off of pneumococcal competence. *Proceedings of the National Academy of Sciences of the United States of America* **110**, E1035-1044, doi:10.1073/pnas.1219868110 (2013).

18 Martin, B., Prudhomme, M., Alloing, G., Granadel, C. & Claverys, J. P. Cross-regulation of competence pheromone production and export in the early control of transformation in Streptococcus pneumoniae. *Molecular microbiology* **38**, 867-878 (2000).

19 Ulrych, A. *et al.* Characterization of pneumococcal Ser/Thr protein phosphatase phpP mutant and identification of a novel PhpP substrate, putative RNA binding protein Jag. *BMC microbiology* **16**, 247, doi:10.1186/s12866-016-0865-6 (2016).

20 Stamsas, G. A. *et al.* Identification of EloR (Spr1851) as a regulator of cell elongation in Streptococcus pneumoniae. *Molecular microbiology*, doi:10.1111/mmi.13748 (2017).

21 Zeghouf, M. *et al.* Sequential Peptide Affinity (SPA) system for the identification of mammalian and bacterial protein complexes. *Journal of proteome research* **3**, 463-468 (2004).
22 Lecointe, F. *et al.* Anticipating chromosomal replication fork arrest: SSB targets repair DNA helicases to active forks. *The EMBO journal* **26**, 4239-4251, doi:10.1038/sj.emboj.7601848 (2007).
23 Fleurie, A. *et al.* Mutational dissection of the S/T-kinase StkP reveals crucial roles in cell division of Streptococcus pneumoniae. *Molecular microbiology* **83**, 746-758, doi:10.1111/j.1365-2958.2011.07962.x (2012).
24 de Jong, I. G., Beilharz, K., Kuipers, O. P. & Veening, J. W. Live Cell Imaging of Bacillus subtilis and Streptococcus pneumoniae using Automated Time-lapse Microscopy. *Journal of visualized experiments : JoVE*, doi:10.3791/3145 (2011).
25 Lefevre, J. C., Claverys, J. P. & Sicard, A. M. Donor deoxyribonucleic acid length and marker effect in pneumococcal transformation. *Journal of bacteriology* **138**, 80-86 (1979).
26 Mortier-Barriere, I., de Saizieu, A., Claverys, J. P. & Martin, B. Competence-specific induction of recA is required for full recombination proficiency during transformation in Streptococcus pneumoniae. *Molecular microbiology* **27**, 159-170 (1998).
27 Chastanet, A., Prudhomme, M., Claverys, J. P. & Msadek, T. Regulation of Streptococcus pneumoniae clp genes and their role in competence development and stress survival. *Journal of bacteriology* **183**, 7295-7307, doi:10.1128/JB.183.24.7295-7307.2001 (2001).
28 Dagkessamanskaia, A. *et al.* Interconnection of competence, stress and CiaR regulons in Streptococcus pneumoniae: competence triggers stationary phase autolysis of ciaR mutant cells. *Molecular microbiology* **51**, 1071-1086 (2004).
29 Martin, B. *et al.* ComE/ComE~P interplay dictates activation or extinction status of pneumococcal X-state (competence). *Molecular microbiology* **87**, 394-411, doi:10.1111/mmi.12104 (2013).
30 Berge, M. J. *et al.* Midcell recruitment of the DNA uptake and virulence nuclease, EndA, for pneumococcal transformation. *PLoS pathogens* **9**, e1003596, doi:10.1371/journal.ppat.1003596 (2013).
31 Fleurie, A. *et al.* MapZ marks the division sites and positions FtsZ rings in Streptococcus pneumoniae. *Nature* **516**, 259-262, doi:10.1038/nature13966 (2014).
32 Kausmally, L., Johnsborg, O., Lunde, M., Knutsen, E. & Havarstein, L. S. Choline-binding protein D (CbpD) in Streptococcus pneumoniae is essential for competence-induced cell lysis. *Journal of bacteriology* **187**, 4338-4345, doi:10.1128/JB.187.13.4338-4345.2005 (2005).
33 Akerley, B. J. *et al.* Systematic identification of essential genes by in vitro mariner mutagenesis. *Proceedings of the National Academy of Sciences of the United States of America* **95**, 8927-8932 (1998).
34 Caymaris, S. *et al.* The global nutritional regulator CodY is an essential protein in the human pathogen Streptococcus pneumoniae. *Molecular microbiology* **78**, 344-360 (2010).
35 Johnston, C. *et al.* RecFOR is not required for pneumococcal transformation but together with XerS for resolution of chromosome dimers frequently formed in the process. *PLoS genetics* **11**, e1004934, doi:10.1371/journal.pgen.1004934 (2015).
36 Le Bourgeois, P. *et al.* The unconventional Xer recombination machinery of Streptococci/Lactococci. *PLoS genetics* **3**, e117, doi:10.1371/journal.pgen.0030117 (2007).
